# Forecasting the Impact of Knowledge of International Humanitarian Law Acquired by Ukrainian Medical Students on Their Practical Activities and the Formation of Humanity in Society

**DOI:** 10.15388/Amed.2026.33.1.12

**Published:** 2026-07-22

**Authors:** Oksana Soroka, Volodymyr Voloshynovych, Yulia Kotsyubynska

**Affiliations:** 1 Ivano-Frankivsk National Medical University, Ivano-Frankivsk, Ukraine

**Keywords:** armed conflict, medical education, humanism, international humanitarian law, medical students, Ukraine, ginkluotas konfliktas, medicinos mokymas, humanizmas, tarptautinė humanitarinė teisė, medicinos studentai, Ukraina

## Abstract

**Purpose:**

International humanitarian law (IHL) and the principle of medical neutrality are vital for safeguarding human life and dignity during armed conflicts. This study aimed to evaluate the current state of IHL education in Ukrainian medical institutions and forecast its impact on future healthcare professionals’ professional practice and humanistic orientation.

**Methods:**

A mixed-methods design was applied, including content analysis of curricula from 17 higher education institutions across different ministries and an online sociological survey of 174 respondents, primarily medical students and healthcare professionals. Quantitative data were analyzed by using frequency distributions and Spearman correlation, while qualitative analysis explored the respondents’ ethical reasoning in conflict-related scenarios.

**Results:**

Only one-third of Ukrainian medical universities included dedicated IHL courses, primarily as electives. Self-reported awareness of IHL was low to moderate, with 23% rating their knowledge as good and 26% as poor. Despite this, 81.4% of medical students agreed with impartial medical assistance to all victims, regardless of affiliation. A weak but positive correlation (ρ=0.15) was found between IHL knowledge and humanitarian attitudes. Female students demonstrated stronger humanitarian orientations compared to males.

**Conclusion:**

IHL education in Ukraine remains fragmented and insufficient, yet students display a high ethical potential aligned with humanitarian principles. Structured, value-oriented IHL curricula, including practical training and case-based learning, are needed to strengthen medical neutrality, enhance ethical decision-making, and foster the formation of humanistic values in Ukrainian society.

## Abstract (it)

**Tikslas:**

Tarptautinė humanitarinė teisė (THT) ir medicininio neutralumo principas yra gyvybiškai svarbūs siekiant apsaugoti žmonių gyvybę ir orumą ginkluotų konfliktų metu. Šio tyrimo tikslas buvo įvertinti dabartinę THT mokymo būklę Ukrainos medicinos įstaigose ir prognozuoti jos poveikį būsimų sveikatos priežiūros specialistų profesinei praktikai bei humanistinei orientacijai.

**Metodai:**

Buvo taikytas mišrus tyrimo dizainas, įskaitant 17 skirtingoms ministerijoms pavaldžių aukštųjų mokyklų mokymo planų turinio analizę ir internetinę sociologinę apklausą, kurioje dalyvavo 174 respondentai, daugiausia medicinos studentai ir sveikatos priežiūros specialistai. Kiekybiniai duomenys analizuoti naudojant dažnių pasiskirstymus ir Spearmano koreliaciją, o pasitelkus kokybinę analizę tirtas respondentų etinis samprotavimas konfliktinių situacijų kontekste.

**Rezultatai:**

Tik trečdalyje Ukrainos medicinos universitetų buvo įtraukti specialūs THT kursai, daugiausia kaip pasirenkamieji dalykai. Savarankiškai vertinamas THT žinojimas buvo nuo žemo iki vidutinio lygio: 23 % respondentų savo žinias įvertino kaip geras, o 26 % – kaip prastas. Nepaisant to, 81,4 % medicinos studentų sutiko, kad medicinos pagalba turi būti teikiama visiems nukentėjusiems, neatsižvelgiant į jų priklausomybę. Nustatyta silpna, bet teigiama THT žinių ir humanitarinių nuostatų koreliacija (ρ = 0,15). Studentės parodė stipresnę humanistinę orientaciją nei studentai.

**Išvada:**

THT mokymas Ukrainoje išlieka fragmentinis ir nepakankamas, tačiau studentai demonstruoja didelį etinį potencialą, atitinkantį humanitarinius principus. Siekiant sustiprinti medicininį neutralumą, pagerinti etinį sprendimų priėmimą ir skatinti humanistinių vertybių formavimąsi Ukrainos visuomenėje, būtina sukurti struktūruotas, vertybėmis grįstas THT programas, įtraukiant praktinį mokymą ir atvejo analizę.

## Introduction

In the contemporary context of armed conflicts and humanitarian crises, *International Humanitarian Law* (IHL) has gained particular significance, especially for medical professionals who often find themselves on the frontline of events. In Ukraine, which has been facing Russian armed aggression since 2014, the application of IHL and the principle of medical neutrality have become not only theoretical principles but also practical instruments for protecting human life and dignity [1].

Medical professionals working in conflict zones are confronted daily with ethical dilemmas associated with assisting all those affected, regardless of their affiliation. Within this context, knowledge of IHL becomes an integral component of the professional training of future physicians, underscoring the necessity of incorporating these competencies into the curricula of higher medical education institutions [2].

This study aims to analyze the current state of international humanitarian law education in Ukrainian higher education institutions, particularly within medical specialties, and to assess the impact of such knowledge on the development of professional and humanistic qualities among future physicians. The article also examines the correlation between the level of medical students’ awareness of IHL and their ethical orientations, particularly regarding the provision of medical care under conditions of an armed conflict.

This relevance of this research lies in its theoretical contribution to the advancement of medical education and its practical importance for shaping the ethical consciousness of future professionals who will operate under armed conflict and emergency circumstances. The findings may serve as a foundation for subsequent reforms in the medical training system, aimed at strengthening humanistic values and professional standards in healthcare.

## Methods

This mixed-methods cross-sectional study combined historical review, curriculum content analysis, and a sociological online survey. The study was based on a comprehensive approach which combined a historical analysis of the development of international humanitarian law (IHL), an examination of educational documentation, and a sociological survey. The historical dimension covered the evolution of IHL from the first Geneva Conventions of the 19^th^ century to modern standards, particularly in protecting medical personnel during armed conflicts. This made it possible to trace the transformation of the principles of medical neutrality and their influence on contemporary curricula.

The core of the study consisted of official teaching materials from 17 higher education institutions in Ukraine, operating under the jurisdiction of various ministries. In particular, syllabi, academic curricula, and working programs from 10 universities under the Ministry of Education and Science of Ukraine, as well as from 7 institutions within the systems of the Ministry of Defence, Ministry of Justice, Ministry of Internal Affairs, and Ministry of Health, were analyzed. All materials were obtained from the official websites of these institutions. The curricula and syllabi were collected from official university websites between March and April 2025.

The analysis of the curricula employed content analysis and comparative analysis methods. Special attention was given to the following aspects: the presence of courses on IHL in academic plans (while distinguishing between compulsory and elective courses), the specifics of their titles, and the degree of integration of IHL into non-core specialties. Comparative analysis enabled the identification of differences in teaching approaches to IHL across various educational sectors.

Another critical component of the research was a sociological survey involving 174 respondents. The sample included medical students (over 50%), physicians, law students, and representatives of other groups. The survey was conducted online from January to June 2025. Quantitative analysis included frequency distributions and Spearman correlation with the objective to assess relationships between IHL knowledge, ethical orientations, and attitudes toward justice. Qualitative analysis interpreted open-ended responses to explore moral dilemmas and practical implications of applying IHL in conflict situations. Quantitative analyses were performed by using *Excel Office 365*. The study also explicitly considered instructional hours, the course status (compulsory vs. elective), and the inclusion of practical exercises and case studies in the analysis of curricula.

Such a sample was designed to meet the study’s core objective, that of examining the impact of IHL knowledge primarily on future and current medical professionals, while preserving the possibility of comparison with other target groups. The survey was conducted online; it included questions aimed at assessing the respondents’ awareness of the fundamental principles of IHL and identifying their ethical orientations.

Both quantitative and qualitative methods of data analysis were applied. Quantitative analysis included frequency distributions and the identification of correlations between the level of IHL knowledge and the respondents’ ethical views. Qualitative analysis enabled a deeper interpretation of the participants’ open-ended comments concerning ethical dilemmas related to applying IHL norms.

Based on the data obtained, a forecast was developed regarding the impact of IHL knowledge on the professional activities of future medical practitioners and the development of their personal qualities.

Some limitations of the study should be acknowledged. These include the predominance of medical students in the sample, which may affect the representativeness of the results, as well as the lack of detailed information in some instances about the number of hours allocated for IHL instruction in curricula. Nevertheless, the combination of different research methods provided a comprehensive picture of the state of IHL teaching in Ukrainian higher education and its influence on the development of professional and personal qualities of future specialists.

All respondents were informed about the study objectives, and participation was completely voluntary. The responses were anonymized, and data integrity was ensured through cross-verification and double-entry checks. Ethical approval was not required for this study.

## Results

### International Humanitarian Law and Medical Neutrality: Historical and Legal Aspects

International humanitarian law constitutes a component of public international law. It regulates the conduct of armed conflicts and protects persons who do not, or no longer, participate in hostilities. Its core principles – distinction, proportionality, humanity, and necessity – possess legal and profound ethical foundations, particularly in medical practice [3].

IHL (the law of armed conflict), which comes into force with the onset of an armed conflict or peacekeeping operation, is a system of internationally recognized legal norms and principles applicable during armed conflicts. These norms establish the rights and obligations of subjects of international law, restrict or prohibit the use of specific means and methods of warfare, ensure the protection of conflict victims, and define liability for violations. The primary objective of IHL is the protection of persons not directly participating in hostilities, as well as those who have ceased to do so due to illness, injury, or other reasons, irrespective of race, skin color, political or religious beliefs, gender, ethnic or social origin, property status, place of residence, language, or any other characteristics [4].

The origins of modern IHL can be traced back to the war for Italian independence in 1859, which had devastating humanitarian consequences. The large-scale loss of life and the brutality of the First and Second World Wars underscored the necessity of establishing effective international instruments and mechanisms for human rights protection. Among the most important international treaties are the four Geneva Conventions of 12 August 1949 on the protection of war victims: the Convention for the Amelioration of the Condition of the Wounded and Sick in Armed Forces in the Field; the Convention for the Amelioration of the Condition of Wounded, Sick and Shipwrecked Members of Armed Forces at Sea; the Convention relative to the Treatment of Prisoners of War; and the Convention relative to the Protection of Civilian Persons in Time of War. Additional Protocols to these Conventions include Protocol I (Protection of Victims of International Armed Conflicts), Protocol II (Protection of Victims of Non-International Armed Conflicts), and Protocol III (Adoption of an Additional Distinctive Emblem). Together, these treaties form the so-called ‘Geneva Law’, which focuses on protecting victims of war. By contrast, the ‘Hague Law’ primarily addresses restrictions on means and methods of warfare, and includes instruments such as the 1907 Hague Convention on the Laws and Customs of War on Land, the 1954 Convention for the Protection of Cultural Property in the Event of Armed Conflict, the 1972 Biological Weapons Convention, the 1980 Convention on Certain Conventional Weapons, the 1993 Chemical Weapons Convention, and the 1997 Ottawa Treaty on Anti-Personnel Mines [5].

The first Geneva Convention, regarded as the starting point of modern IHL, codified the principle of non-interference with medical personnel and the inviolability of medical facilities, later formalized as ‘medical neutrality’. Armed conflicts, however, have posed serious challenges to this principle, as medical staff and facilities have frequently been targeted, and prisoners of war or the sick have been killed. These realities prompted the adoption of the aforementioned Additional Protocols, which strengthened the protection of medical personnel and facilities.

The history of IHL in general, and of medical neutrality in particular, is the history of a struggle for humanism and respect for human life. Contemporary challenges, such as international terrorism, global conflicts, and the ongoing Russian armed aggression against Ukraine, highlight the urgent need for IHL education, which is becoming an integral part of 21^st^-century education, serving as a mechanism for ensuring survival in times of armed conflict. Therefore, disseminating IHL knowledge is not only a responsibility of the state and public authorities but also an objective necessity for society as a whole [6].

### Specific Aspects of Teaching International Humanitarian Law in Ukrainian Educational Institutions

Overall, the field of IHL as a branch of international law has been thoroughly elaborated, in contrast to the issues of teaching and instruction in IHL within educational institutions. Our analysis of these aspects has demonstrated the following.

What concerns secondary education institutions, in 2022, the Ministry of Education and Science of Ukraine disseminated methodological materials to the regions concerning the integration of IHL into courses on history, civic education, and law in schools and institutions of postgraduate pedagogical education. In the view of the authors, the dissemination of IHL knowledge and the development of civic competences among pupils and educators on its basis constitute a critically important step for fostering both societal and individual awareness of the necessity of applying IHL norms in the context of the armed aggression of the Russian Federation against Ukraine. Understanding the inadmissibility of war crimes and the inevitability of legal accountability for their commission, cultivating models of safe behavior for children and adults under conditions of armed conflict, and creating prerequisites for the reintegration of temporarily occupied territories are essential educational objectives for the school system of Ukraine [7]. IHL-related content has been integrated into history, civic education, and law school curricula per the newly developed methodological guidelines and revised educational programs.

As for higher education institutions under the authority of the Ministry of Education and Science of Ukraine, our analysis of the available teaching materials (syllabi, curricula, and working programs) published on official university websites reveals that IHL is currently taught across various Ukrainian universities, which testifies to the recognition of its importance [8]. In most cases, however, IHL is offered as an elective course, which may indicate insufficient institutionalization within curricula. In many universities, IHL is not a firmly established and compulsory part of the educational process; its study often depends on the student’s choice or the institutional opportunities of a given university. Only in a few institutions is IHL compulsory for students majoring in “International Law”, underlining its significance within this particular discipline. Variability in course titles is also observed, including “International Humanitarian Law”, “Law of Armed Conflict”, and “Human Rights Protection during Armed Conflicts”, thereby reflecting differing teaching approaches and emphases on particular aspects of IHL. The discipline is taught at Bachelor’s and Master’s levels, thus covering multiple tiers of higher education. Moreover, IHL is taught within the “Law” specialization and fields such as “International Relations”, thus highlighting its interdisciplinary character.

Unlike institutions under the Ministry of Education and Science, universities administered by other ministries – specifically, those under the Ministry of Defence, the Ministry of Justice, the Ministry of Internal Affairs, and the Ministry of Health – exhibit a sector-specific approach to IHL instruction, oriented toward the needs of their respective domains: military affairs, penitentiary systems, law enforcement, and healthcare. IHL is taught across all higher education levels, from Bachelor’s to Master’s and doctoral programs. The specializations include “Law”, “Medicine”, and “Law Enforcement”, thus emphasizing the relevance of IHL for professionals in diverse sectors, particularly those related to security, conflict management, and human rights protection. In these institutions, IHL is also predominantly offered as an elective, which indicates that, despite its recognized importance, it is not consistently a mandatory component of study programs. Course titles often reflect the applied nature of instruction, such as “Legal Status of Military Personnel and the Protection of Human Rights and Freedoms during Armed Conflicts”, “Application of IHL Norms in the Activities of Penal Institutions”, and “International Human Rights Standards in Law Enforcement”, underscoring the adaptation of IHL content to the specific professional needs of each sector.

At the same time, in higher military educational institutions under the Ministry of Defence of Ukraine, our analysis of teaching materials available on official websites indicates that the subject “International Humanitarian Law” is currently not included in their curricula. However, IHL instruction is stipulated in NATO standards, which now guide the reform of Ukraine’s higher military education system. In particular, NATO Standard STANAG 2449 outlines principles of training in the law of armed conflict and the objectives of such training. In 2019, STANAG 2449 was updated with the educational publication “Law of Armed Conflict Training”, which defines the relevant learning and teaching principles, while emphasizing that training in the law of armed conflict must be integrated at all levels of military education, including individual and collective training [9]. This process is currently underway, with the implementation of so-called legal tracks combining several study directions, including military legislation, IHL, the legal status of military personnel, and the protection of human rights and freedoms during armed conflicts.

In medical higher education institutions under the Ministry of Health of Ukraine, specific features related to the particularities of the medical profession and the role of healthcare personnel in armed conflicts were identified. IHL is not widely represented in their curricula; however, selected issues are integrated into law, military medicine, healthcare organization, and medical ethics courses. Some medical universities offer courses such as “Medical Law” or “Medical Jurisprudence”, which may cover, *inter alia*, aspects of protecting healthcare personnel and facilities in times of armed conflict. However, teaching medical neutrality as a separate subject is not common.

Analyzing IHL teaching materials in Ukrainian educational institutions has allowed us to conclude that strengths and areas for improvement have been identified. A positive trend can be observed toward strengthening IHL teaching and diversity in program structures and content. Nonetheless, there remains a need for unification and adaptation to contemporary challenges, as well as for enhancing faculty expertise in IHL and engaging international organizations and experts in cooperation. Significant contributions in this regard are made by organizations such as the Ukrainian Helsinki Human Rights Union and the Ukrainian Red Cross Society, whose activities aim to raise public awareness – particularly among military personnel, law enforcement officers, healthcare workers, journalists, and volunteers – of IHL norms and the protection of individuals affected by armed conflicts. Key educational resources in this area include:

- The online course for educators on the Prometheus platform, “Studying International Humanitarian Law: An Online Course for Educators” – which has been developed by a group of practitioner-experts from the Ukrainian Helsinki Human Rights Union and the Ukrainian Red Cross Society with the support of the Ministry of Education and Science of Ukraine [10];
- comprehensive educational visual materials on IHL for schoolchildren, including videos, presentations, and tests [11];
- a compilation of IHL programs for teacher training in postgraduate pedagogical education institutions [12];
- the educational manual “Studying International Humanitarian Law”, aimed at acquiring basic IHL knowledge, enhancing legal and civic competences, communication skills, curiosity, responsibility, and a holistic view of armed conflict [13];
- the information note “The Role of National Societies during International Armed Conflicts within the Framework of IHL: A Working Review” outlines and explains the role of the Red Cross and Red Crescent National Societies during international armed conflicts under IHL, including in situations of occupation, even those established without armed resistance [14].

### Forecast of the Impact of Knowledge of International Humanitarian Law Acquired by Ukrainian Medical Students on Their Practical Activities and the Formation of Humanity in Society

In the ongoing armed conflict in Ukraine, fostering humanitarian values among medical students has become particularly urgent. During war, medical professionals face frontline ethical dilemmas, and knowledge of International Humanitarian Law (IHL) can serve as an intellectual and moral compass, enabling them to maintain professionalism, neutrality, and humanity under extreme conditions [15].

The authors conducted a survey aimed at assessing the level of awareness and the formation of ethical orientations among young Ukrainian professionals and current students regarding IHL and its application in armed conflict situations, with a subsequent forecast of how this knowledge might influence their future practical activities and the development of humanitarian qualities. Specifically, the study sought to determine how well the respondents (and particularly medical students) understand the basic principles of IHL; whether medical workers and students support key humanitarian principles such as neutrality and impartial assistance to victims of armed conflicts; and to explore the respondents’ moral attitudes toward justice and responsibility, which indirectly affect their perception of humanity and human rights. The collected data may serve as evidence supporting the need to integrate or strengthen IHL teaching in medical and other educational programs, especially considering armed conflicts in Ukraine and worldwide. Based on current knowledge and attitudes, the study aimed to forecast how acquisition of more in-depth IHL knowledge might affect medical professionals’ practical activities (including ethical decision-making and safety) and the development of personal qualities (empathy, impartiality, responsibility).

The authors performed both quantitative and qualitative analyses of responses to key survey questions, including:

- Respondent status: to filter the target group;
- Age and gender: to understand the demographic profile of the respondents.
- Awareness of basic IHL principles: to determine the level of IHL knowledge;
- Agreement or disagreement with the statement “Medical professionals should assist all victims of armed conflict between opposing parties, regardless of their affiliation”: to assess adherence to humanitarian principles;
- The choice of a justice system is 1) where the innocent may be punished but the guilty can never escape punishment, 2) where the innocent will never be punished but the guilty may escape, and 3) other: to determine ethical and value orientations.

A Spearman correlation analysis was conducted to identify statistical relationships between IHL knowledge, humanitarian orientations, and the choice of justice system.

The results showed a weak but positive correlation between IHL knowledge and humanitarian orientation (ρ = 0.15), indicating the potential role of IHL education in shaping the students’ ethical attitudes. At the same time, no significant correlation was found between humanity and the choice of a justice system (ρ = -0.03), nor between IHL knowledge and the choice of a justice model (ρ = 0.04), thus suggesting the autonomy of legal perceptions from the level of humanitarian expertise.

The analysis of the collected data allows the following conclusions:

The target audience – i.e., medical students – represented the largest group with 91 respondents (approximately 52.3% of the total sample); other groups included dental students (7), doctors (26), other medical professionals (4), lecturers (4), law students (10), lawyers (2), and others (10). The respondents’ age was as follows: the vast majority (169 of 174) were 18–29 years old, which is expected for a student population. The distribution of gender was: 114 women (65.5%), 59 men (33.9%), one not specified, thus reflecting the general gender trends in medical education institutions.

Cross-analysis also revealed differences in humanitarian orientations depending on the respondents’ social status and gender (Table 1). The highest mean scores for agreement with the principle of providing medical care to all victims were observed among female dental students (4.57), female medical students (4.37), and other female medical professionals (4.50). In contrast, the scores were lower among male lecturers and doctors (3.50 and 3.88, respectively). This may indicate higher ethical sensitivity among young women when forming their professional identity.

### Awareness of International Humanitarian Law among the Respondents

The respondents’ awareness of International Humanitarian Law (IHL) was predominantly moderate or low: excellent – 2 (2.2%); good – 21 (23.1%); satisfactory – 39 (42.9%); poor – 24 (26.4%); unable to assess – 5 (5.5%). These results indicate a heterogeneous level of knowledge and underscore the need to strengthen the teaching of IHL in higher medical education institutions. Even a ‘satisfactory’ level may be insufficient to apply IHL principles in crises effectively.

Regarding the necessity to provide medical assistance to all victims during armed conflicts irrespective of their affiliation, the attitudes among medical students were as follows: fully agree – 33 (36.3%); rather agree – 41 (45.1%); somewhat disagree – 13 (14.3%); disagree entirely – 3 (3.3%); unable to assess – 1 (1.1%). This indicates that most medical students (81.4% = 36.3% + 45.1%) fully agree with assisting all affected individuals. This highly significant indicator demonstrates a strong moral and ethical recognition of a key humanitarian principle. Nevertheless, the presence of 17.6% (14.3% + 3.3%) of students who ‘rather disagree’ or ‘completely disagree’, as well as those unable to decide (1.1%), highlights gaps in understanding the universality of medical ethics in an armed conflict. This critical group of 18.7% represents individuals for whom IHL knowledge could substantially influence their attitudes.

Regarding ethical perspectives on medical assistance, 106 respondents agreed that medical personnel should assist all victims regardless of their side in the conflict, whereas 58 respondents partially or fully disagreed. These findings demonstrate a degree of ethical division, indicating the need for additional discussion and training on medical ethics in the context of an armed conflict.

### Choice of Legal System

Eighty respondents supported a stricter system where “an innocent person may be punished, but the guilty will never escape punishment”; 57 respondents preferred a more humane system where “an innocent person will never be punished, but the guilty may escape punishment”; a relatively large portion (37) considered alternative options necessary. This suggests that young people tend to favor legal systems emphasizing the punishment of the guilty, even at the risk of unjust conviction. This may indicate insufficient understanding of justice values or underlying moral dilemmas.

The potential correlation between survey responses regarding the choice of a legal system and medical students’ attitudes was analyzed. Medical students represent an influential societal group which will shape the future trajectory of social relations, trust in state institutions, and the overall level of humanism.

The choice of a system where “an innocent person may be punished, but the guilty will never escape punishment” prioritizes the inevitability of punishment for the guilty, even at the risk of harming the innocent. Under such circumstances, society may gravitate toward stricter norms, increased surveillance, and oversight, while citizens might feel ‘protected’ from criminality, yet simultaneously experience elevated state pressure. The state-citizen relations may adopt an authoritarian character, where security and public order precede individual freedoms and guarantees. Societal suspicion may rise, as the presumption of innocence is not absolute, and public condemnation may precede legal proceedings. Humanism may be weakened in terms of protecting the rights of the accused, with the focus shifting from rehabilitation and crime prevention to punitive measures. In a conflict context, this system can generate additional ethical dilemmas for healthcare professionals, where pressure to quickly ‘identify enemies’ may conflict with medical ethics and IHL principles regarding assisting all in need, not only to ‘one’s own’.

Conversely, choosing a system where “an innocent person will never be punished, but the guilty may escape punishment” prioritizes the protection of innocence, even at the risk of impunity for the guilty. In this scenario, society will likely strengthen the protection of individual rights, freedoms, and the presumption of innocence. The state is perceived as a guarantor of human rights rather than a potentially repressive apparatus. Social relations are likely to be more open and tolerant, with citizens enjoying strong safeguards against unjust accusation. Trust in the system increases, underpinned by the principle of “better to let ten guilty go than punish one innocent”, demonstrating adherence to fundamental human rights and dignity. Humanism and humanity become cornerstones of societal relations and justice, fostering empathy, rehabilitation, and understanding complex circumstances. Such a system aligns with medical ethics and IHL principles, as both prioritize life, dignity, and protection for all in need, without discrimination.

The respondents choosing ‘other’ (aspiring to an ideal system where “an innocent person will never be punished, and the guilty will never escape punishment”) envision a dynamic society, with the state functioning as an effective protector and a just punisher, and citizens as active participants in legal processes. This vision reflects deep humanism, valuing individual dignity and collective justice, promoting empathy, responsibility, and high moral standards. In such a society, medical ethics and IHL principles are integral to social values, maximally supporting professional practice.

Thus, a societal preference for punishing the guilty at any cost, while even risking harm to the innocent, may foster a more authoritarian, less free and humane society. Conversely, prioritizing the protection of the innocent promotes a society with greater freedom, robust human rights, and profound humanism. Aspirations toward an ideal balance indicate a mature society seeking the perfection of the rule of law, high trust, harmonious social relations, and maximized humanistic values.

The finding that nearly 50% of medical students selected the system protecting the innocent is a highly positive signal, indicating that future Ukrainian healthcare professionals possess a strong humanistic potential and a commitment to living in a society that values human rights. This value orientation will likely be naturally integrated into their professional practice, fostering humanity and contributing to constructing a culture aligned with the highest standards of humanism and democracy. Therefore, the choice of a legal system reflects not merely an abstract opinion but deep-seated values that, when scaled to the societal level, will inevitably shape future development.

### Potential Impact of IHL Knowledge on Humanistic Orientation

The survey results also revealed significant indicators of the potential impact of IHL knowledge on the formation of humanistic orientations among medical students. Notably, the indicator of ethical readiness to act within IHL principles is highly significant: 39 respondents fully agreed, and an additional 56 ‘rather agreed’ with the statement on the necessity to provide medical assistance to all victims regardless of their affiliation in the conflict.

This approach demonstrates the predominance of the principle of humanity – which is a core provision of the 1949 Geneva Conventions – over political or ethnic biases [16].

These findings support a justified prediction that IHL knowledge fosters moral resilience and humanistic thinking among students, even with limited legal awareness. Similar conclusions are consistent with other studies demonstrating that even basic familiarity with IHL promotes empathy, responsibility, and ethical self-regulation in future healthcare professionals [17].

Although only four students rated their IHL knowledge as ‘very good’, and the majority (73 respondents) assessed it as ‘satisfactory’, the existing level of humanistic orientation constitutes a substantial resource for developing ethical readiness. The observed imbalance between theoretical knowledge and personal convictions (manifested in 53 respondents supporting a judicial system in which the guilty may escape punishment to protect the innocent) underscores the need for an interdisciplinary approach to IHL education, incorporating ethical, legal, and clinical components.

Simultaneously, the students’ preference for legal models minimizing the risk of punishing the innocent indicates a prevailing humanistic paradigm grounded in natural law concepts and aligned with the principles of medical ethics [18]. This suggests that comprehensive, value-oriented IHL education will serve as legal instruction and a mechanism for fostering enduring motivation toward humane behavior under professional challenges, including war-related ones.

Forecasting this impact is further supported by the students’ willingness to engage in ethical discussions of real wartime scenarios. The development of moral resilience – i.e., the capacity to maintain ethical orientation under extreme stress, resource scarcity, or pressure – requires formal IHL instruction and integrating case analysis, simulations, training, and interdisciplinary interaction with legal experts, psychologists, and clergy. Such engagement can facilitate more profound reflection on ethical and humanistic principles, enhancing internal motivation for humane conduct, enabling students to appreciate human dignity, and supporting ethical decision-making under complex war conditions. This approach aligns with the recommendations of the International Committee of the Red Cross, which emphasize that “IHL education should be not only formal but also value-oriented, fostering an internalized need to act humanely” [19].

## Discussion

This study revealed substantial discrepancies in teaching International Humanitarian Law (IHL) across different segments of higher education in Ukraine. The most systematic integration of IHL was observed in institutions under the Ministry of Defence and the Ministry of Justice, where it is a mandatory part of professional training. This finding is consistent with the professional context of these institutions, as their graduates are directly involved in armed conflict-related activities.

However, integrating IHL into medical education was found to be insufficient, as only 33% of medical higher education institutions included dedicated IHL courses in their curricula. This result contradicts international recommendations, including the Geneva Conventions, which stress the crucial role of healthcare professionals in protecting the rights of individuals affected by armed conflict. Compared with other studies emphasizing the centrality of IHL in medical training, the present findings highlight an urgent need for reform in the Ukrainian medical curricula.

Another significant result is the weak association between self-assessed IHL knowledge and readiness to act according to humanitarian principles. The observed correlation (ρ = 0.15) suggests that knowledge alone is insufficient to ensure the development of humanistic attitudes. This supports the view that education should be complemented by value-oriented pedagogical strategies to transform theoretical knowledge into practical ethical readiness.

Gender- and status-related differences were also observed. Female students demonstrated higher ethical readiness to assist regardless of context, indicating strong humanistic potential within this group. By contrast, male students and practicing physicians showed relatively lower indicators, suggesting that targeted educational interventions may be necessary. Similar patterns have been reported in other studies exploring gender differences in ethical decision-making, thus reinforcing the need for differentiated approaches to IHL teaching.

This study has several limitations. First, the findings are based on self-reported data, which may be subject to bias. Second, the analysis was limited to a sample of Ukrainian higher education institutions, and therefore, the results may not be generalizable to all contexts. Third, the cross-sectional design does not allow for causal conclusions about the relationship between IHL knowledge and ethical attitudes.

Future research should include longitudinal studies to assess the long-term impact of IHL education on professional behavior and expand the scope of analysis to other countries to enable cross-national comparisons. It would also be valuable to explore qualitative dimensions, such as students’ reflections and case-based experiences, to better understand how IHL education shapes ethical consciousness.

In conclusion, despite the limited integration of IHL into the Ukrainian medical curricula, this study demonstrates the high potential of students to internalize principles of humanity, neutrality, and professional responsibility. Incorporating IHL and medical neutrality into medical education could strengthen the ethical foundations of healthcare, enhance collaboration with international partners, and prepare professionals capable of delivering quality care in conflict and emergency settings. These findings highlight the educational value of IHL for promoting humanistic attitudes and safeguarding medical ethics in times of war.

## Conclusions

1. Only one-third of Ukrainian medical higher education institutions include dedicated courses on International Humanitarian Law, which indicates insufficient attention to this critically important subject.
2. Awareness of IHL among medical students remains unsatisfactory – as 23% rate their knowledge as good, while 26% consider it poor – and exerts only a limited positive influence on humanistic orientations (ρ = 0.15, Spearman correlation).
3. A direct relationship exists between IHL knowledge and readiness to adhere to medical ethics principles as students with higher levels of IHL knowledge are 23% more likely to support an impartial approach to patient care. However, the correlation coefficient indicates a weak strength of this relationship (ρ = 0.15).
4. Current IHL curricula for medical students have significant shortcomings, including insufficient instructional hours (12–16) along with a lack of practical case studies and simulations.
5. To address these gaps, a mandatory IHL course should be introduced, emphasizing practical application and interdisciplinary learning, adapted to the conditions of armed conflict.
6. The projected impact of IHL knowledge on the development of humanity and humanistic values among Ukrainian medical students is positive, provided a comprehensive, value-oriented educational approach is implemented, as supported by empirical statistical analysis.
